# “We are in this together:” dyadic-level influence and decision-making among HIV serodiscordant couples in Tanzania receiving access to PrEP

**DOI:** 10.1186/s12889-021-10707-x

**Published:** 2021-04-14

**Authors:** Virginia A. Fonner, Jacob Ntogwisangu, Isihaka Hamidu, Juliet Joseph, Joshua Fields, Evans Evans, Jordan Kilewo, Claire Bailey, Lloyd Goldsamt, Celia B. Fisher, Kevin R. O’Reilly, Theonest Ruta, Jessie Mbwambo, Michael D. Sweat

**Affiliations:** 1 Medical University of South Carolina, Department of Psychiatry and Behavioral Sciences, Division of Global and Community Health, Charleston, SC USA; 2 Department of Psychiatry and Mental Health, Muhimbili University of Health and Allied Sciences, Dar es Salaam, Tanzania; 3 Medical University of South Carolina, College of Medicine, Charleston, SC USA; 4 New York University, Rory Meyers College of Nursing, New York, NY USA; 5 Fordham University, Department of Psychology and Center for Ethics Education, Bronx, NY USA

**Keywords:** HIV, Pre-exposure prophylaxis, Dyad, Serodiscordant couple, Relational autonomy, Qualitative research

## Abstract

**Background:**

A substantial number of new HIV infections in sub-Saharan Africa occur within stable couples. Biomedical prevention (pre-exposure prophylaxis, PrEP) and treatment (antiretroviral therapy, ART) can provide benefits to sexual partners and can be used to prevent infection within HIV serodiscordant couples. However, research is typically focused on individuals, not dyads, even when the intervention may directly or indirectly impact sexual partners. Gaps remain in understanding best practices for recruitment, informed consent, and intervention implementation in studies involving HIV prevention and treatment among heterosexual serodiscordant couples. This qualitative study was undertaken to understand and describe decision-making and dyadic-level influence among members of serodiscordant couples regarding (1) participation in a dyadic-based research study involving HIV self-testing and access to PrEP, and (2) utilization of PrEP and ART.

**Methods:**

This qualitative study was nested within an observational cohort study assessing the acceptability of home-based couples’ HIV self-testing and uptake of dyadic care for serodiscordant couples involving facilitated referral for HIV-positive partners and access to PrEP for HIV-negative partners. Semi-structured in-depth interviews were conducted among a subset of study participants (*n* = 22) as well as individuals involved in serodiscordant relationships who chose not to participate (*n* = 9). Interviews focused on couples’ decision-making regarding study participation and dyadic-level influence on medication use. Interviews were transcribed verbatim and translated from Kiswahili into English. Data were analyzed using thematic analysis.

**Results:**

Three major themes were identified: (1) HIV as “two people’s secret” and the elevated role of partner support in serodiscordant relationships; (2) the intersectional role of HIV-status and gender on decision-making; (3) the relational benefits of PrEP, including psychosocial benefits for the couple that extend beyond prevention.

**Conclusions:**

The study found that couples made joint decisions regarding study participation and uptake of HIV-related medication. Relational autonomy and dyadic-level influence should be considered within research and programs involving HIV serodiscordant couples.

**Supplementary Information:**

The online version contains supplementary material available at 10.1186/s12889-021-10707-x.

## Background

An estimated 1.6 million people are living with HIV in Tanzania, [1] and heterosexual transmission remains the dominant mode of HIV transmission in the region. Modelling suggests that close to 60% of new HIV infections in sub-Saharan Africa occur within stable couples [2]. Research has found rates of HIV seroconversion within married couples in Tanzania are higher than within the general population, with incidence rates among married women significantly greater than for married men [3]. This finding reflects the significant gender differences regarding HIV infection and risk seen across sub-Saharan Africa, [4, 5] with girls and women disproportionately affected due to biological, social, cultural, and economic factors [6–9].

Advances in effective biomedical prevention for HIV, including pre-exposure prophylaxis (PrEP) [10–12] and recognition of the inability to transmit HIV once virally suppressed [13, 14] as notably recognized through the global U=U campaign (Undetectable [viral load] = Untransmittable), [15] have altered the HIV prevention landscape. Choices for prevention are expanding for serodiscordant couples, but their effectiveness may depend on couples’ ability to take coordinated action regarding uptake and adherence.

Several PrEP and early antiretroviral therapy (ART) trials and demonstration projects have signaled that partner support is a major consideration for study participation and medication adherence [16–18]. For example, two placebo-controlled PrEP trials among women in sub-Saharan Africa, FEM-PrEP and VOICE, found PrEP to be ineffective in preventing HIV but also found low overall drug adherence [19, 20]. Follow-up interviews with participants found that decisions were strongly influenced by perceived support or disapproval from partners, families, and communities [21, 22]. Research among heterosexual couples in sub-Saharan Africa have found several distinct patterns of partner influence on ART adherence—both positive (e.g., providing support) and negative (e.g., control and conflict)—that are informed by relationship quality, power dynamics, and structural factors [23, 24].

Despite these examples of partner influence on uptake of and adherence to HIV prevention and treatment, decision-making is traditionally conceptualized as an individual process [25]. Autonomy, defined as an individual’s capacity for self-determination (i.e., having freedom and agency to make decisions), [26] serves as a core principle of bioethics and is also primarily conceptualized individually. As most life decisions are influenced by our social context, particularly influence from a partner or other significant relationship, gaps exist in our understanding of how to incorporate and allow for dyadic-level influence in research and programs while still promoting autonomy among couples [27]. This is especially true for interventions requiring dyadic participation, or for those directly affecting both members of a couple, thus requiring some level of joint decision making.

The concept of relational autonomy, which values and acknowledges the social influences and interdependence that realistically inform decision-making, [27, 28] is particularly salient for research involving HIV. Although HIV prevention and treatment are most frequently conceptualized individually, these decisions are often made within dyadic relationships. Research has shown that taking protective measures against HIV, such as using condoms, can create mistrust between partners or serve as a tacit admission of infidelity, thus pitting love and trust against prevention [29–31]. Similarly, conversations about HIV and HIV testing, especially among long-term stable couples, have been described as an “unusual event” signifying extraordinary circumstances, such as sickness or discovery of an extramarital affair, and not a matter for routine discussion [32]. Couple-based interventions for HIV prevention have demonstrated that strengthening interpersonal communication can lead to increased HIV testing and reduced sexual risk behavior [33–35]. These findings, along with a growing body of research, [13, 23, 24, 33–42] demonstrate the importance of dyadic-level influence on HIV prevention and treatment.

Relational autonomy is particularly relevant to HIV because methods of prevention and treatment are uniquely intertwined as the actions of one person may directly affect another. The term “treatment altruism” has been used to describe motivations of people living with HIV to prevent transmission to sexual partners, [43, 44] such as by choosing to use condoms or start ART specifically to benefit the uninfected partner [18]. Additionally, PrEP has given people an alternative to barrier-style prevention methods, including condom use and abstinence, which has served to empower couples in serodiscordant relationships through increasing sexual intimacy and trust, decreasing stigma, and regaining a sense of normalcy for couples’ sex lives [16, 45, 46]. A qualitative study regarding serodiscordant couples’ decisions on whether to continue PrEP during pregnancy within a study in Kenya and Uganda concluded that using a relational autonomy approach could potentially simultaneously advance women’s autonomy while still allowing for male partner engagement [17].

Additionally, gender roles and norms dictate how men and women interact in heterosexual relationships. Women often have less power to negotiate in sexual relationships as sexual-decision making is male-dominated, [47] have less access to education and resources, [48] and often face physical and sexual violence [7, 49]. Men, conversely, often adhere to dominant, or “hegemonic,” masculinities, which refer to patterns of practices that assert men’s power over women [50]. As related to HIV risk, these masculinities often manifest themselves through high-risk sexual behavior, engaging in age-disparate relationships, intimate partner violence, and transactional sex [7, 49, 51, 52]. These factors all play a role in shaping HIV-related risk, as well as influence partner communication and decision-making within couples.

Given the expanding HIV prevention options for heterosexual serodiscordant couples that leverage the interconnectedness between prevention and treatment, it is critical to understand how serodiscordant couples make decisions to protect their own health and that of their partner(s), and it is equally critical to understand how to honor and appropriately involve both members of couples in research studies affecting them. Although research has demonstrated dyadic-level influence in decision-making regarding uptake of ART and PrEP when viewed as separate interventions among HIV serodiscordant couples in sub-Saharan Africa, [23, 24, 36, 37, 41, 42] to our knowledge no study has examined decision-making among couples who were offered dyadic enrollment in a research study involving access to both PrEP and ART simultaneously. To fill this gap, we conducted a qualitative study among HIV serodiscordant couples in Tanzania to understand dyadic-based decision-making as relevant to 1) participation in a research study providing access to PrEP and referral to ART and 2) uptake of HIV-related prevention (PrEP) and treatment (ART).

## Methods

### Study context

This qualitative sub-study was nested within a larger observational cohort study taking place in Kisarawe, Tanzania to examine the feasibility, safety, and impact of a dyadic-based HIV self-testing and dyadic care component for serodiscordant couples. Specifically, the parent study intervention included door-to-door provision of HIV self-testing kits to stable cohabitating couples, and a dyadic-based prevention and care component (hereto referred to as “dyadic care”), which offered access to PrEP for HIV-negative members of serodiscordant couples and referral to treatment (ART) for HIV-positive members. Couples who learned of their serodiscordance through self-testing and couples who already knew of their serodiscordance were eligible to enroll in the dyadic care portion of the study. To enroll in either part of the study (self-testing or dyadic care), couple agreement and consent for dyadic enrollment was required, although individuals could continue study participation even if their partners subsequently dropped out or were lost to follow-up. As this study sought to understand couples’ decision-making surrounding research participation, we interviewed members of serodiscordant couples who chose to participate in dyadic care as well as those who were eligible but chose not to participate.

### Participant recruitment

Participants were purposively sampled, with maximum variation sought for gender and HIV status. For individuals currently enrolled in dyadic care, potential participants were approached and offered enrollment in the qualitative sub-study immediately following completion of their regularly scheduled parent study research appointments (e.g., either 6- or 12-months following enrollment). Participants who participated in self-testing but chose not to participate in dyadic care were approached immediately following a 6-month home-based post-self-testing follow-up visit. To recruit known serodiscordant couples identified through the HIV Care and Treatment Center (CTC) who chose not to participate in dyadic care, individuals were approached at the CTC by study staff following regularly scheduled appointments. During recruitment for the qualitative sub-study, both members of a couple were eligible to participate, although dyadic participation was not required.

### Semi-structured interviews

In-depth interviews lasting thirty minutes to one hour were conducted with each participant. All interviews were conducted one-on-one, and interviewees were assured their responses would be kept confidential. If both members of a couple agreed to participate, interviews took place separately but simultaneously. Topics covered in the interviews included: (1) history of HIV testing, partner disclosure, and process of serodiscordance revelation (i.e., partners finding out they have different HIV statuses), (2) decision-making process regarding study participation/non-participation and decision-making within households more generally; and (3) uptake of PrEP and/or ART, including any partner influence on uptake or discontinuation. Interviews with current participants in dyadic care focused on all three areas. Interviews with non-participants focused mainly on the process of partner disclosure, serodiscordance revelation, and exploring reasons for non-participation. Interview guides specific to each sub-population involved were developed and are presented in Additional file 1.

Interviews took place at the study center, or a location of the participant’s choosing. All interviews were conducted in Kiswahili by one of three trained research assistants (two men and one woman). When possible, interviewers were gender-matched to the gender of interviewees. Interviews were audiotaped, transcribed verbatim, and translated from Kiswahili to English by translators fluent in both languages, frequently by the same interviewer who conducted the interview. Participants received 10,000 Tanzanian shillings (approximately $5 U.S.) for their time. All participants provided written informed consent.

### Data analysis

During data collection regular team debrief meetings were held to assess emerging themes and adapt interview guides accordingly. Following data collection, given the importance of cultural context in understanding gender dynamics, data were iteratively analyzed using both a cultural insider (emic) and outsider (etic) perspective, which is a technique previously employed in Tanzania to comprehensively understand and interpret qualitative data across diverse cultural backgrounds [53]. The cultural insiders included Tanzanian qualitative researchers who were fluent in Kiswahili and English, and the cultural outsiders included a team of U.S.-based researchers experienced in qualitative research. To ensure critical meaning was not lost in translation, the Tanzanian team read through the original transcripts in Kiswahili whereas the U.S.-based team read the English translations. Both teams wrote extensive summaries and detailed memos about each interview. Discrepancies, questions, and cultural significance between the Kiswahili and English versions were accounted for and discussed. Data were coded (using the English translations) in Atlas.ti (Version 8) by members of the U.S. and Tanzanian teams, using a coding structure informed by salient concepts derived from the initial analysis. Both deductive and inductive codes were used to develop the hierarchical coding structure guided by the initial emic and etic group discussions.

Concepts from the constant comparative method [54] were used to compare within interviews, across sub-groups (e.g., comparing males versus females, HIV-positive versus HIV-negative, participants versus non-participants, etc.) and across individuals within a couple, if both members of the couple were interviewed. This technique has been successfully used with dyadic data, [55] and allowed for the articulation of a model that integrates different perspectives to understand dyadic-level influence on study participation and uptake of study interventions.

### Ethical statement

The study received ethical approvals from the institutional review boards at the Medical University of South Carolina, and Muhimbili University of Health and Allied Sciences. The study was also approved by the Tanzanian National Institute of Medical Research.

## Results

We conducted interviews with 31 individuals involved in HIV serodiscordant relationships, including 22 current dyadic care participants and nine non-participants. Of the non-participants, six were recruited from the self-testing component of the study and three from the CTC. Overall the mean age was 47.9 years (range: 32–71). The sample included eight HIV-negative men (26%), six HIV-positive men (19%), four HIV-negative women (13%), and 13 HIV-positive women (42%). In five cases, both members of a couple chose to participate, including two serodiscordant couples recruited from the self-testing component who chose not to participate in dyadic care and three serodiscordant couples who participated in dyadic care. Of the five serodiscordant couples in which both members participated, each member of the couple presented similar and complimentary descriptions of discussions they had engaged in about study participation and decisions regarding intervention uptake.

Three interrelated themes were identified: (1) HIV serodiscordance as “two people’s secret” and the elevated role of partner support in serodiscordant relationships; (2) The role of HIV status and gender in dyadic decision-making; (3) the relational benefits of PrEP and the dyadic-focused intervention more generally. The first two themes were applicable to current participants in dyadic care as well as non-participants. Of all non-participants interviewed, most had communicated with the partner about the study. Any differences between dyadic care participants and non-participants are included in the discussion of the specific themes.

### Theme 1: Serodiscordance as “two people’s secret” and the elevated role of partner support in serodiscordant relationships

HIV-positive participants mainly reported limited disclosure of their HIV status. In these cases, revelation of serodiscordance was typically shared only between members of a couple. As one HIV-positive man described, HIV within his relationship was “two people’s secret.” In some cases, disclosure of HIV was also shared with select family members, with disclosure conferred only to those whom the individual felt were trustworthy. Reasons for limited disclosure mainly concerned anticipated stigma and fear of discrimination. As one HIV-negative woman stated:

“*There is no one in my family that is aware of [my husband’s HIV status]. … They [family members] will tell you that you are stupid, you’re living with a person who is like that. They will have different percept*ions.” *(HIV-negative woman, P12)*

Since HIV serostatus was not widely shared, HIV-positive participants drew support from those who did know—their partners, which elevated the partners’ role in offering and providing HIV-related support. The revelation of serodiscordance often shifted a couple’s perception from thinking of HIV as an individual issue to a couples-based issue, thus impacting subsequent courses of action within their relationships. As one participant stated when reflecting on the course of action taken following revelation of serodiscordance:

*“Because he loves me and I also love him so we decided to live [together], so we love each other and we are living as normal but we are protecting each other.” (HIV-positive woman, P21)*

Reasons for joining the study reflected perceived benefits to oneself, one’s partner, or the couple overall. A desire to protect one’s partner and one’s family was a consistent theme across dyads. As one participant said:

*“That’s why I decided to join in the study, because I thought in the study they will help me not to infect my family.” (HIV-positive man, P16)*

In addition to joining the study to access PrEP, about half of couples reported initiating regular use of condoms during sex following revelation of serodiscordance in order to protect the uninfected partner. The theme of mutual support extended into joint encouragement for medication use for both PrEP and ART, as well as more general support. As one participant stated:

*“I started taking the medication [PrEP], so I encouraged him [my husband], and we take our medication every day.” (HIV-negative woman, P12)*

However, two women reporting medication discontininuation due to partner influence. In one instance, an HIV-positive woman reported stopping ART for ten years because her partner was not supportive:

*“I started the medication and went back home but I had a little conflict with my partner. He told me I am infected so I have to leave the house. I told him that I could not leave the house even though I am taking the medication because I have little kids to look after, I will take my medication while I am here and God will help me, but my partner was aggressive so I stopped taking the medication for 10 years...” (HIV-positive woman, P7*)

In another instance, a woman discontinued PrEP because her HIV-positive partner felt that taking PrEP would encourage her to seek sexual partners outside of their marriage. As she described:

*“But after a few months of taking these pills [PrEP], my husband changed … he insists on thinking that I have another partner, he is telling me that I am careless because I am currently using pills [PrEP]... That is why I reached a point where I decided to stop using these pills” (HIV negative woman, P31)*

### Theme 2: the role of HIV status and gender in dyadic decision-making

Individual-level characteristics, particularly HIV status and gender, influenced HIV-related partner communication, including disclosure and discussions about study participation. In particular, HIV-positive women often experienced more worry and stress leading up to disclosure than HIV-positive men. Women often expected relationship dissolution following revelation of serodiscordance. Upon learning that their partners would not abandon them following disclosure, women expressed relief and happiness, and often felt that these positive responses were atypical and unexpected. As one participant said:

*“In this world most of men and women, if they hear one of them is HIV positive, it is when they will pack and leave you there. Or for men if I was the one diagnosed with HIV he could tell me to go back to my parents. But we were supporting each other although we had different statuses. So we had to plan because we had no way out, and here we are until today.” (HIV-negative woman, P12)*

Gender norms and differences regarding HIV within a couple were well known and discussed among participants. Norms included the acceptable and typical response for partners, especially men, to leave relationships following a partner’s HIV diagnosis, the disinclination of men to test for HIV, and gendered perceptions of HIV. As one participant described:

*“When a woman has HIV people may consider her as a prostitute, it is better if it could be him [a man], as you know, our societies know that when a man goes outside his marriage it is a normal thing, but it is a strange thing if woman does the same”. (HIV-positive woman, P10).*

Regarding household decision-making, many participants (*n* = 9/31)—both men and women—identified the man as the primary household decision-maker. However, more typically men and women reported that practical decision-making came through agreement and partner discussion (*n* = 16/31). In three cases, women reported being primary decision makers for the household. In cases where the man was the reported decision-maker, women did not report feeling disempowered, and some equated a husband’s decision-making with love and support:

*“I feel happy when my husband influences me. I know he cares about me.” (HIV-positive woman, P15)*

Decisions about study participation were made jointly. Members of a couple either learned about the study together, such as learning from a staff member following couples-based self-testing, or one member—the HIV positive partner—learned about the study from the HIV clinic and subsequently relayed the information to their partners. No participant reported feeling coerced to join, but most participants, both men and women, HIV-positive and HIV-negative, reported being encouraged or advised by their partner to join the study. Regardless of who the perceived decision-maker was, participants reported difficulties having HIV-related conversations with their partners. Challenges arose in part due to gender dynamics and power differentials between HIV-positive and HIV-negative partners. In this respect, the study itself (i.e., the door-to-door provision of self-test kits) and study staff, especially counselors, often helped to jumpstart conversations and facilitated difficult conversations following testing and revelation of serodiscordance. As one participant said:

*“It was very easy for me [to discuss joining the study with my partner], but if I would have been the one who started that conversation, it would have been a problem, but luckily after those research people talked to us, it made him [my husband] start the conversation in the evening … ” (HIV-positive woman, P10)*

At the dyadic level, if couples were mutually supportive of each other, communicated with each other about the study, and found the study beneficial to their relationship (as described in the previous theme), they were generally accepting of the intervention.

Among the nine non-participants interviewed, partners still expressed support for one another. The main reasons reported for not joining the dyadic care portion of the study included misunderstandings about who was eligible for dyadic care, miscommunication with study staff, or the long-term absense of partners at the time of study enrollment. One HIV-positive man said that he told his wife about the study but decided not to join because he was too busy and felt it would be a waste of time for his partner. The couple still discussed the study together, and the wife agreed with her husband’s decision not to join. Of note, two individuals who participated in self-testing but did not participate in dyadic care chose not to discuss their HIV status or that of their partner during the interview, thus the interviewer did not ask about their reasons for choosing not to join the dyadic care portion of the study.

### Theme 3: relational benefits of PrEP and the dyadic-based intervention more generally

Almost all participants reported that using PrEP to prevent HIV infection was a primary benefit of study participation, although other psychosocial benefits were commonly discussed. Medication usage, both ART and PrEP, was generally encouraged and supported by each member of the couple. For HIV-positive partners, having PrEP accessible to their HIV-negative partners helped alleviate feelings of guilt and worry about infecting a partner. As one participant said:

*“He [my husband] is not someone who wants to use condom a lot. So even myself I am worried he can be infected, therefore when I heard there are those medicines [PrEP], I considered it as a good thing...” (HIV-positive woman, P10).*

For HIV-negative partners, in addition to the personal preventative benefits from PrEP, participants described taking medication as an act of symbolic solidarity that provided psychological benefits to both members of the couple. As one participant said:

*“After I started using medicine [PrEP], it touched her [my wife] … because she was very happy to see we had reached a point where we using our medicine without any complaints, without questioning each other.” (HIV-negative man, P17)*

All nine participants with access to PrEP as part of the study reported initiating PrEP, and two reported PrEP discontinuation. One woman mentioned discontinuation was directly tied to partner influence as discussed in the previous theme. The other participant reported stopping PrEP because she was not sick and “wanted a rest”. As she described:

*“But I see it is difficult to take medicine for me. Because I ask myself I don’t have HIV infection but I take medicine the same like the one who is sick … I see it is not good at all. I think I carry a heavy burden...all the time it is medicine, medicine, medicine, the same as the sick person. Sometimes it becomes difficult, because sometimes you can say like, I am not sick but I take medicine the same as my partner? It is very difficult”. (HIV-negative woman, P3)*

In this case, the partner living with HIV was supportive of his partner’s decision to stop PrEP because he was receiving treatment. As he explained (P6), “Because she has me, she will not get infected because I have care.”

PrEP, along with the education and counseling couples received as part of the intervention, offered couples a way forward for their relationship following revelation of serodiscordance. As one HIV-positive man (P16) stated, *“maybe I can say that protection [PrEP] gave us hope.”* Participants also spoke frequently of love and commitment as driving forces for PrEP use. As one HIV-negative man (P25) stated, *“It is only love that made me use those medicines [PrEP].”*

Additionally, the education and counseling that participants received as part of the intervention, irrespective of PrEP use, was often cited as the most beneficial aspect of the study, because it taught couples how they could manage their serodiscordance and continue living together. As one participant stated:

*“In short she [my wife] felt like she could not do anything [following disclosure], or our marriage would be dead, but due to the counseling which I received I told her that we will continue living—we will know what to do.” (HIV-negative man, P18)*

Among couples in which condom use was unacceptable, PrEP use seemed essential to maintaining the serodiscordant relationship. As one HIV-positive man (P24) said, *“[Without PrEP], our love will no longer be there in our house.”* An HIV-positive woman (P10) reported similar feelings, stating: *“I see these medicines [PrEP] are the ones to save my marriage.”* Among these participants, there was significant concern about what would happen once the study ended and PrEP was no longer accessible to HIV-negative partners.

## Discussion

This qualitative study was conducted to understand decision-making among couples regarding study participation and PrEP/ART use within an observational cohort study offering self-testing to cohabitating couples and access to PrEP among couples found to be serodiscordant. Our findings suggest that couples who participated in dyadic-based care embraced a relationship-centered approach to HIV and relied on partner support. HIV status and gender dynamics factored into initial disclosure and discussions of study participation, but overall couples reported making decisions jointly. Study operations and study personnel played a critical role in helping to facilitate understanding about serodiscordance and influenced dyadic motivation for study participation and medication use. Couples felt that PrEP had benefits that extended beyond HIV prevention, including psychosocial advantages that helped both members of the couple, as well as the relationship itself. Participants’ understanding of treatment as prevention (i.e., U=U) was less clear, although one couple specifically referenced the protective benefits of consistent ART in making decisions regarding PrEP discontinuation. As PrEP among HIV serodiscordant couples has been proposed as a “bridge” for prevention until the HIV-positive partner is virally suppressed, [56] more research is needed to understand how couples understand and interpret U=U.

Our results resonate with Lewis’s model of interdependence and communal coping as a means to understanding health behavior change [57]. In this model, Lewis posits that when faced with a health threat, couples’ predisposing factors, including individual communication style, demographics, etc., influence their “transformation of motivation,” which entails a reorientation from a self-centered to a relationship-centered approach [57]. Within our study, we found that couples frequently underwent a transformation of motivation following revelation of serodiscordance in terms of understanding HIV as a relationship issue as opposed to an individual one. In Lewis’ theory, following the transformation of motivation, couples engage in communal coping, which involves having a shared belief that working together to address the health threat is advantageous to the relationship. In our study, couples engaged in communal coping by embracing HIV as a relational issue and taking concrete steps, such as joining the study and using PrEP and/or ART, to address it. These results are supported by prior qualitative research that utilized Lewis’s theory to compare communal coping among serodiscordant couples involved in a microbicide trial in Uganda and Zambia and found more evidence of communal coping at the trial site that recruited serodiscordant couples together, as opposed to the site that recruited female partners only [58].

Our results are also in agreement with prior studies focused on examining gender dynamics in terms of PrEP adherence [59] and HIV status disclosure [60] among couples. In terms of adherence, Ware et al. found that among serodiscordant couples in Uganda, PrEP was seen as a solution to relationship viability following the “discordance dilemma” in which partners wanted to stay together but feared infecting the other/becoming infected, [59] which is similar to our findings. In terms of disclosure, Bhatia et al. found that partner communication about HIV among couples in South Africa was generally infrequent and that gender inequalities impacted partners’ ability to disclose, [60] which also aligns with our findings.

Within our study, study staff and the study operations themselves served as a means to overcome issues surrounding partner communication by providing an opportunity (i.e., home-based HIV self-testing and access to PrEP) and facilitator (i.e., guiding conversations) for change. As these interventions were implemented in the context of research, interactions with participants, which included multiple home visits to provide HIV self-test kits and discern use, were most likely more intense than they would be in a programmatic setting. However, given participants’ reliance on and appreciation of study staff for helping facilitate conversations about HIV, especially following HIV self-testing and disclosure, the importance of interactions between staff and participants should not be overlooked when considering programmatic implementation.

Our results demonstrate there are concrete steps studies and programs can take to facilitate dyadic engagement and communication, such as ensuring that if partners learn of the intervention sequentially, the initial partner has sufficient understanding and information to discuss the opportunity with the other partner, including provision of communication tips, and if needed, having a counselor available to facilitate discussions between partners. PrEP and ART programs could directly address partner engagement by providing those in need of PrEP or ART with skills and tools to increase agency and communication within their relationships, as was done with the CHARISMA intervention to enhance uptake of the dapivirine vaginal ring for PrEP among women with regular partners [61, 62].

Our findings also support a relational interpretation of autonomy when working with serodiscordant couples and highlight the importance of considering partner-level influence at all stages of research, including recruitment, informed consent, and implementation. Osamar and Grady suggest gender-informed couples’ joint decision-making should be viewed on a continuum spanning varying degrees of autonomy, [27] with decisions considered autonomous if they are made with intentionality, understanding, and freedom from external control [26]. Although it was challenging to parse out from participant responses as to whether these criteria were satisfied, in most cases participants seemed to be making joint-decisions with clear intentionality and understanding, and external influences from partners mostly aligned with the individual’s and couples’ perceived best interest.

Importantly, within our study members of couples generally described making decisions with their partners and acting based on feelings of mutual respect, love, and trust. Prior research has acknowledged that the subjective experiences of couples in sub-Saharan Africa, specifically involving emotions such as love, are understudied, [63] yet incorporating the emotional dimension of couples’ experiences into interventions has the potential to significantly impact communication patterns and health behavior change [64]. Regarding future interventions in sub-Saharan Africa promoting PrEP and ART adherence among serodiscordant couples, our results suggest interventions that promote partner communication and informed choice of prevention modalities *within a couple* (as opposed to an individual) are promising. Providing couples with tools to promote joint-decision-making while allowing for autonomous choice could be supported by ensuring trained counselors are available to facilitate conversations, providing education on dyadic implications of intervention options (e.g., benefits of PrEP and U=U), and fostering more equitable gender norms, including normalizing partner communication about HIV, at the community level.

### Limitations

Our sample predominantly involved serodiscordant couples who were current participants in the dyadic care portion of the parent study, meaning these were couples who had been tested for HIV, mutually disclosed their HIV results, and made a mutual decision to engage in dyadic care. Therefore, these couples demonstrate a high level of dyadic coordination that may not be representative of couples who either never test for HIV, or never disclose results, or disclose results but have unsupportive partners. This is an important limitation as rates of partner abandonment for HIV-positive members of mutually-disclosed serodiscordant couples remain high, especially for women [65]. We attempted to overcome this limitation by identifying and interviewing eligible couples who were aware of the study but chose not to participate. However, recruitment for this group was challenging and only nine individuals were identified, so it is possible data saturation concerning reasons for non-participation was not achieved. It is also possible we missed views of couples who initially chose to participate but were lost to follow-up over the course of 18-months. Additionally, our findings are likely highly dependent on the sociocultural context and geographic location in which the research occurred. However, given our findings are in agreement with results from prior qualitative studies involving PrEP and serodiscordant couples from other locations in East and Southern Africa, the collective findings suggest a more generalizable model.

## Conclusions

Decisions about HIV testing, HIV prevention, and HIV treatment often take place within a dyadic context, yet interventions and perceptions of autonomy remain mostly focused on the individual. Findings from this study demonstrate that some individuals in serodiscordant relationships are making decisions related to HIV prevention and treatment based not only on perceptions of what is best for them as individuals, but also what is best for their relationships and their partners. Taking into account dyadic-level influence and acknowledging relational autonomy is needed in HIV-related research and programs involving serodiscordant couples.

## Supplementary Information

**Additional file 1.** Interview guides. This file contains English versions of all three interview guides used in this study. The first interview guide pertains to individuals who are current participants in the parent study; all of whom are members of HIV serodiscordant couples. The second interview guide pertains to individuals involved in serodiscordant relationships who were approached about joining the parent study at the Care and Treatment Center (the local HIV clinic) but who chose not to join the parent study. The third interview guide pertains to individuals involved in serodiscordant relationships who participated in the self-testing phase of the parent study but chose not to enroll in the dyadic care portion of the study (which provided access to PrEP for the HIV-negative partner and referral for ART for the HIV-positive partner).

## Data Availability

The datasets generated and analysed during the current study are not publicly available due to the sensitive nature of information collected from participants regarding HIV. Qualitative interview transcripts are only visible to the direct research team.
