# Supplementary material for: “We are in this together:” dyadic-level influence and decision-making among HIV serodiscordant couples in Tanzania receiving access to PrEP
Source: BMC Public Health. 2021 Apr 14;21:720. doi: 10.1186/s12889-021-10707-x (PMC8045366; doi:10.1186/s12889-021-10707-x)
Supplement: Supplementary file 1 — Additional file 1. Interview guides. This file contains English versions of all three interview guides used in this study. The first interview guide pertains to individuals who are current participants in the parent study; all of whom are members of HIV serodiscordant couples. The second interview guide pertains to individuals involved in serodiscordant relationships who were approached about joining the parent study at the Care and Treatment Center (the local HIV clinic) but who chose not to join the parent study. The third interview guide pertains to individuals involved in serodiscordant relationships who participated in the self-testing phase of the parent study but chose not to enroll in the dyadic care portion of the study (which provided access to PrEP for the HIV-negative partner and referral for ART for the HIV-positive partner). [file 12889_2021_10707_MOESM1_ESM.docx]

# Additional File 1

## Interview Guide #1: Current participants in the parent study (Dyadic-based Diagnosis, Care, and Prevention Study, known as “Wenza Huru” in Swahili)

**Interview guide for Current DDCP participants**

Date of Interview: __ __/ __ __/__ __

Person conducting interview: ______________________________

Participant ID: __ ___ ___

***Introduction [to be read by interviewer]:*** *Thank you for agreeing to share your experiences with me about your participation in Wenza Huru. I am going to be asking you about your experiences with HIV testing and finding out your HIV status, joining the Wenza Huru Study, and about your experiences with the study.*

**Part A: Interview Questions**

**History of HIV testing and knowledge of HIV status**

1. To start off with, please tell me about the time you first considered getting tested for HIV?
   1. Probes: What made you want to get tested? What role did your partner play?
2. Please tell me about the time when you realized you had a different HIV status than your partner?
   1. Probes: How did you talk about it with your partner? How did you feel? What changed in your relationship? What steps did you take, if any, to prevent transmission of HIV?

**Decision Making about Participation and the Informed Consent Process**

1. Please tell me how you first heard about the Wenza Huru study?
2. Please tell me about your reasons for wanting to join the Wenza Huru study?
3. Please tell me the reasons why you think your partner wanted to join the study?
4. Please tell me about the conversations you had with your partner about joining the study
   1. Probes: Who brought it up? Any disagreements? Felt pressured by partner? When did conversations take place? Who usually makes decisions in your relationship? How do you feel about this?
5. How did you decide as a couple to join the Wenza Huru study?
6. How did you feel about joining the Wenza Huru study as a couple (as opposed to as an individual)?
7. What were the benefits to you for joining the Wenza Huru study? Your partner? Your relationship?
8. What were the risks to you for joining the Wenza Huru study? Your partner? Your relationship?
9. What role, if any, did any other family members (besides your partner) play in your decision to join the study? What about your community (e.g. worried about stigma)?
10. How is research perceived in this community?
11. Before joining the study, you met with study staff and were walked through the process of informed consent [show old consent form; remind participants of the process]. We are going to walk through this process, and please tell me what you remember about each step and how you felt about each step. *[The steps are: 1) provided information about the study, 2) asked whether you would be interested in joining, given time to discuss as a couple, 3) separated for signing consent form, and 4) brought back together to begin enrollment]. The interview should remind the participant about each of these steps and pause to ask what the participant remembers about each step and how they felt during each step.*
    1. Probes: Did you feel you could decline participation if you wanted to? What do you think should have been done differently at each step? How was your partner involved in this process?
12. What if you had wanted to participate and your partner did not want to participate. What would you have done?
    1. Probe: What if it was the other way around—you did not want to participate but your partner did?

**Relevant to PrEP/ART use**

1. What questions or concerns did you have about PrEP when you joined the study? About ART?
   1. Concerns for yourself? Concerns for your partner?
2. What benefits did you see to using PrEP when you joined the study? Benefits to ART?
   1. Probes: Benefits to you? Benefits to your partner? To others?
3. How did you decide what you and your partner were going to do [regarding using study drugs]?
   1. Probes: Who brought up the PrEP/ART? What were your concerns? Your partners? Did you feel pressure to take a drug? Pressure to not take a drug?
4. How did taking/not taking the drugs affect the relationship with your partner?
5. How did your partner’s decision to take PrEP or ART affect your decision to take PrEP or ART?
6. Since you enrolled and made your initial decision, have you or your partner made a change to your PrEP/ ART use (example: decided to stop using PrEP)?
   1. Probes: If so, what prompted the change? Has your partner made a change? If so, what prompted the change? How did you make the decision? What role did your partner play?
7. In the future, what do you think will happen once the study is over and you may not have access to PrEP?
   1. Probes: How will this affect your relationship? Your health?
8. Is there anything else about your study experience that you would like to discuss?
9. How could we make this study better for you and your partner?

***[END OF QUALITATIVE INTERIVEW]:*** *Thank you for taking the time to complete this interview. Before we finish, I am going to ask you a few questions about yourself.*

Demographic Information:

Gender:

Age:

Education:
Occupation:

HIV status:

HIV status of partner:

Participation in Wenza Huru (choose one):

(1) Self-testing ONLY

(2) Self-testing and dyadic care

(3) Dyadic care ONLY

(4) No participation in study but was informed about the study and invited to join

## Interview Guide #2: Members of HIV serodiscordant couples who were approached at the Care and Treatment Center (CTC) but who chose not to join the parent study

**Interview guide for couples approached at CTC who chose not to join study**

Date of Interview: __ __/ __ __/__ __

Person conducting interview: ______________________________

Participant ID: __ ___ ___

***Introduction [to be read by interviewer]:*** *Thank you for agreeing to share your experiences with me about your knowledge and thoughts about the Wenza Huru Study. I am going to be asking you about your experiences with HIV testing and finding out your HIV status and your thoughts about joining or not joining the Wenza Huru Study.*

**Part A: Interview Questions**

**History of HIV testing and knowledge of HIV status**

1. To start off with, please tell me about the time you first considered getting tested for HIV?
   1. Probes: What made you want to get tested? What role did your partner play?
2. Please tell me about the time when you realized you had a different HIV status than your partner?
   1. Probes: How did you talk about it with your partner? How did you feel? What changed in your relationship? What steps did you take, if any, to prevent transmission of HIV?

**Rationale for deciding to join/not join the Wenza Huru Study**

1. Please tell me how you heard about the Wenza Huru Study?
2. Please tell me what you remember hearing about the Wenza Huru Study? [*after participant answers, provide more complete description of the study to inform remainder of interview*]
3. Please tell me any benefits you can think of receiving from joining the study?
   1. Probes: Possible benefits to you? Possible benefits to partner? Benefits to your relationship? Benefits to others?
4. Please tell me any reasons why you would not want to join the study?
   1. Probes: Concerns for you? Concerns for your partner? Concerns for your relationship? Concern for others? Barriers to you? Barriers to your partner? Other barriers?
5. Please tell me about any conversations you had with your partner about joining the study
   1. Probes: Who brought it up? Any disagreements? Felt pressured by partner? When did conversations take place? Who usually makes decisions in your relationship? How do you feel about this?
6. How did you decide as a couple not to join the Wenza Huru study?
7. What role, if any, did any other family members (besides your partner) play in your decision not to join the study? What about your community (e.g. worried about stigma)?
8. What, if anything, would have made you change your mind about participating?
9. If PrEP were offered at the health clinic outside of the context of research, would you or your partner be interested in taking it? Why or why not?
10. How is research perceived in this community?
11. What if you had wanted to participate and your partner did not want to participate. What would you have done?
    1. Probe: What if it was the other way around—you did not want to participate but your partner did?

***[END OF QUALITATIVE INTERIVEW]:*** *Thank you for taking the time to complete this interview. Before we finish, I am going to ask you a few questions about yourself.*

Demographic Information:

Gender:

Age:

Education:
Occupation:

HIV status:

HIV status of partner:

Participation in Wenza Huru (choose one):

(1) Self-testing ONLY

(2) Self-testing and dyadic care

(3) Dyadic care ONLY

(4) No participation in study but was informed about the study and invited to join

## Interview Guide #3: Members of HIV serodiscordant couples who participated in the self-testing phase of the study but chose not to join the dyadic care portion of the parent study

**Interview guide for self-testing participants**

Date of Interview: __ __/ __ __/__ __

Person conducting interview: ______________________________

Participant ID: __ ___ ___

***Introduction [to be read by interviewer]:*** *Thank you for agreeing to share your experiences with me about your participation in the self-testing phase of Wenza Huru. I am going to be asking you about your experiences with HIV testing and finding out your HIV status, deciding to participate in the study with your partner, and about your experiences with the self-testing.*

**Part A: Interview Questions**

**History of HIV testing and knowledge of HIV status**

1. To start off with, please tell me about the time you first considered getting tested for HIV?
   1. Probes: What made you want to get tested? What role did your partner play?
2. When study staff from Wenza Huru approached you about self-testing for HIV, what were your initial thoughts?

**Decision Making about Participation and the Informed Consent Process**

1. Please tell me about your reasons for wanting to join the self-testing phase of the Wenza Huru study?
2. Please tell me the reasons why you think your partner wanted to join the study?
3. Please tell me about the conversations you had with your partner about joining the study
   1. Probes: Who brought it up? Any disagreements? Felt pressured by partner? When did conversations take place? Who usually makes decisions in your relationship? How do you feel about this?
4. How did you decide as a couple to join the self-testing part of the Wenza Huru study?
5. How did you feel about joining the Wenza Huru study as a couple?
6. What were the benefits to you for joining the Wenza Huru study? Your partner? Your relationship?
7. What were the risks to you for joining the Wenza Huru study? Your partner? Your relationship?
8. What role, if any, did any other family members (besides your partner) play in your decision to join the study? What about your community (e.g. worried about stigma)?
9. How is research perceived in this community?
10. Before joining the study, you met with study staff and were walked through the process of informed consent [show old consent form; remind participants of the process]. We are going to walk through this process, and please tell me what you remember about each step and how you felt about each step. *[The steps are: 1) provided information about the study, 2) asked whether you would be interested in joining, given time to discuss as a couple, 3) separated for signing consent form, and 4) brought back together to begin enrollment]. The interview should remind the participant about each of these steps and pause to ask what the participant remembers about each step and how they felt during each step.*
    1. Probes: Did you feel you could decline participation if you wanted to? What do you think should have been done differently? How was your partner involved in this process?
11. What if you had wanted to participate and your partner did not want to participate. What would you have done?
    1. Probe: What if it was the other way around—you did not want to participate but your partner did?

**Relevant to self-testing**

1. What questions or concerns did you have about self-testing prior to using them?
   1. Concerns for yourself? Concerns for your partner?
2. What benefits did you see to using the self-testing kits when you joined the study?
   1. Probes: Benefits to you? Benefits to your partner? To others?
3. How did you decide what you and your partner were going to do regarding self-testing?
   1. Probes: Who brought up the self-tests? Did you feel pressure to use them?
4. How did using/not using the self-test kits affect the relationship with your partner?
5. How did your partner’s decision to use or not use the self-test kits affect your decision to use or not use the self-test kits?
6. If you used the self-test kit, how did you find the experience?
7. How did you discuss the results of your self-test kit with your partner, if at all?
   1. Probes: What was partner’s reaction? Did partner share his/her results with you?
8. Are you willing to share the results of your HIV self-test for you and your partner with me? *[If yes and response is serodiscordant, proceed with entire interview]. If the participant does not want to share the results, or reports serocondordant results (both partners HIV-negative or both HIV-positive), please proceed to the next questions (Q9-Q11 and the demographic questions), then end the interview.* ***DO NOT PROCEED TO THE FINAL SECTION OF THE INTERVIE****)]*
9. In the future, do you think self-test kits should be made available to people in Tanzania?
   1. Probes: How should they be distributed? What should they cost? How should people who test HIV-positive be linked to care?
10. Is there anything else about your study experience that you would like to discuss?
11. How could we make this study better for you and your partner?

**Relevant to decision to join the second phase of Wenza Huru (ONLY IF SERODISCORDANT)**

1. Thank you for sharing the results of your HIV self-testing with me for you and your partner. During the second visit from the Wenza Huru staff after self-testing, do you recall being told about a second part of the study for serodiscordant couples that involves being able to access pre-exposure prophylaxis (PrEP) to help prevent HIV transmission from the HIV-positive person to the HIV-negative person? *[If the answer is no, conclude the interview and ask demographic questions. If answer is yes, proceed]*
2. What do you recall being told about the second part of the study?
   1. Probes: Who was eligible? What did enrollment require? Enrolled as individual vs. couple?
3. What did you think about this additional study?
   1. Probes: Potential risks to you? To partner? Potential benefits to you? To Partner? What others would think?
4. How did you decide whether to pursue participation in the second part of the study?
   1. Probes: Discussions with partner?
5. If you chose not to participate in the second part of the study involving PrEP, what, if anything, would have helped change your mind?
   1. Probes:

***[END OF QUALITATIVE INTERIVEW]:*** *Thank you for taking the time to complete this interview. Before we finish, I am going to ask you a few questions about yourself.*

Demographic Information:

Gender:

Age:

Education:
Occupation:

HIV status:

HIV status of partner:

Participation in Wenza Huru (choose one):

(1) Self-testing ONLY

(2) Self-testing and dyadic care

(3) Dyadic care ONLY

(4) No participation in study but was informed about the study and invited to join
